# Book Reviews

**Published:** 1803-12-01

**Authors:** 


### CRITICAL ANALYSIS

OF THE

### RECENT PUBLICATIONS

ON THE

### DIFFERENT BRANCHES OF PHYSIC, SURGERY, AND MEDICAL PHILOSOPHY.

---

*The Elements of Physiology; containing an Explanation of the Functions of the Human Body, in which the modern Improvements in Chemistry, Galvanism, and other Sciences, are applied to explain the Actions of the Animal Economy. Translated from the French of* A. Richerand, *by* Robert Kerrison, *Member of the Royal College of Surgeons, London.* Octavo, pp. 464. London, 1803.

A want of some Elementary Treatise on Physiology, taking up the subject where the illustrious Haller has left it, seems to be pretty generally felt; but however alluring to those inclined to take up the pen, such a compendium may appear, the profound research and exhaustive learning of a Haller are easier to be admired than imitated.

The author of the work before us has, however, undertaken the task of imitation, and the present publication is designed as the *Primæ Lineæ* of Physiology, as it now appears.

To do the author justice, he speaks with becoming modesty of his task, when put in comparison with that of his great predecessor; and the Preface to the work before us, is adapted to bespeak the candour and good-will of his readers. Indeed, he appears to consider the nature of his task in somewhat of a different manner to that of the great author of the Elementa Physiologiæ, who declares, " Quando vero ad hunc librum scribendum accessi, dudum in physiologicis laboribus majorem vitæ meæ partem posueram. Neque ergo ignarus adveni magni quod mihi imponebam oneris; felix, si perinde gerendo suffecero." Let us hear what our author says.

"If it should be objected that the enterprize I have undertaken is greater than becomes a person of my age, I shall answer, at the risk of maintaining a paradox, that young men are, perhaps, the most proper for the compilation of elementary works, because their memory retains with more correctness the difficulties they had met with in prosecuting the study, the tracks they had followed to surmount them, and a recent experience makes them acquainted with the defects and advantages of different methods; so that he who in the shortest space of time has acquired the greatest stock of solid information, will be, in many respects, the most able to direct his successors in the thorny paths of instruction and knowledge."

If the composition of an elementary treatise be little more than an affair of memory, if it be only to abridge the lectures of some one favourite teacher, to arrange the contents of a few approved class books, or to attempt to methodize the crude ideas of juvenile disputants, then indeed a young student, a very tyro, possessed of a greater share than usual of sense and industry, may be pronounced competent to initiate his juniors into the intricate paths of science.

It is rather remarkable that, in the present instance, the author really does not do himself justice in this respect, for, though in the execution of his task it is easy to observe that the number of authorities which he has consulted is not very great, yet the work is upon the whole very well executed, full of the necessary information, and not abounding with frivolous or irrevelant matter; so that the author must both have had very good teachers, and must have profited largely from the advantages of education.

In the introductory chapter, which is but confusedly drawn up, the author attempts to explain the animal functions at large, and their general regulating laws. The vital power (which he defines happily enough by a common French expression, the *tout ensemble* of laws that influence the animal economy) he branches out into the two principles of *sensibility* and *contractility*, to which he annexes *caloricity*, or the power in the living body to maintain an equilibrium of temperature. Nothing worthy of remark occurs here, except that the author attempts to prove a *latent* or *imperceptible* sensibility in organs non-sentient in their natural state.

A page or two suffices to discuss the difficult question of the theory of inflammation.

This is his definition, "Inflammation, in my opinion, may be defined, *an augmentation of all the vital powers of that part which is the seat of it.* Sensibility there becomes more lively, mobility greater; and from this increase of sensibility and motion arise all the symptoms that denote the inflammatory state: thus pain, swelling, redness, ness, heat, and the change of secretions, indicate a more energetic and active life in the inflamed part."

To this the following very singular observation is added, "Is not the swelling of an inflamed part induced by the same mechanism as that belonging to parts susceptible of erection, as the *corpora cavernosa* of the penis and clitoris, the nipple, the iris, &c.? In erection of the penis, as in inflammation, there is irritation, afflux of humours to the part, increase of sensibility and motion: yet it is not a state of inflammation. Nature has so disposed the organization of these parts as to permit them, with impunity, to experience this sudden augmentation of vital energy, necessary for the execution of those functions for which the organs they belong to are destined; and like inflammation, these swellings subside when the irritating cause ceases to act."

We may call this a singular illustration and confusion of ideas, for by acknowledging that this local influx of humours is not inflammation, but a very different thing, the hinge on which the similitude hung is at once snapped asunder.

He proceeds to elucidate his arrangement. We need not explain it to our readers, it is sufficiently clear for all useful purposes; for indeed the leading divisions of physiology fall so naturally into classification, that for the mere purpose of methodizing instruction to the beginner, who must go through the whole sooner or later, nice arrangement is but of small importance.

A description of the system of the great sympathetic nerves is added, as forming a nervous system in some degree distinct from the cerebral nerves, and peculiarly intended to give motion and vitality to the internal, assimilating, and digestive functions. This is a very useful view of the subject, and happily introduced here.

To proceed to the body of the work before us, in which however we shall only pause on the parts which appear to require or to merit notice; for the Elements of Physiology are not proper subjects for our Analysis.

The first chapter contains the function of digestion, which is treated in a very perspicuous manner, and the author shews his good sense in confining himself chiefly to matter of fact. The following will give a specimen of the execution of the work:

"It is in the stomach that the mechanism of digestion is principally effected: it has always been considered the most important organ. The aliment received into its cavity becomes more fluid, undergoes a great alteration, and is converted into a soft homogeneous paste, known by the name of chyme. What are the means that induce this change? or, in other words, what is digestion in the stomach?

"It would be useless to recapitulate the hypotheses successively formed to explain digestion; they may be reduced to *coction*, *fermentation*, *trituration*, *putrefaction*, and *maceration* of the food received into the cavity of the stomach. Physiologists are generally agreed at present, in considering digestion in the stomach as a solution tion of the aliment by the gastric juice. This liquid, copiously poured on the internal surface of the stomach when this viscus is irritated by the presence of food, is the production of arterial exhalation; it is neither acid nor alkali, and seems to be of a nature nearly analogous to saliva; the gastric juice, possessing great properties of solution, penetrates into the alimentary matter on all sides, separates and divides its particles, combines with it, changes its composition, and impresses qualities very different from those it possessed before this mixture. In fact, if a mouthful of wine or food be returned from the stomach some minutes after it has been received, the odour, taste, and all the qualities, both physical and chemical, of these substances, are so altered, that we can with difficulty distinguish them; and vinous liquors, more or less acid, are no longer susceptible of spirituous fermentation. The energy of the power of the gastric juice, perhaps exaggerated by some physiologists, is sufficient to reduce to a soft mass the hardest bones, on which certain animals subsist: it is very probable that its chemical composition is different and variable, and that it is acid, alkaline, or saponaceous, according to the nature of the aliment. Although gastric juice is the most powerful agent of digestion in the stomach, its dissolvent power has need of assistance from the action of several secondary causes, as heat, which seems to augment and concentrate itself in the epigastric region. So long as the exertion of the stomach continues, there is a sort of intestine fermentation, which should not be, in the full sense, compared to the motion by which fermentative and putrescent substances are decomposed; there is also a moderate and peristaltic motion of the muscular fibres of the stomach, which press the aliment on all sides, and perform a slight trituration, while the gastric moisture softens and macerates the food before it is dissolved; it may then be affirmed that the process of digestion is, at the same time, chemical, vital, and mechanical: the authors, therefore, of various theories to explain this function have erred by attributing to one cause only, as heat, fermentation, putrefaction, trituration, maceration, and the gastric juice, that which is the aggregate result of all those causes united."

At the conclusion of this chapter a due degree of attention is paid to the chemical analysis of urine, as it is an excretion which on many accounts peculiarly merits the notice of the chemist, and has actually engaged it to very great advantage. It is particularly in the animal chemistry that we may look for the most copious additions to Haller.

Absorption is the subject of the second chapter. The author properly throws some doubts on aqueous absorption from the skin in common circumstances, a question still to be determined by future observation. Somewhat more might have been said on the anatomical distribution of these vessels, and on the assistance which pathology gives in explaining their functions.

The circulation of the blood is treated of in the third chapter. Scarcely any thing occurs here not to be found in Haller, and some interesting interesting points of view are omitted. The effect of pressure, the resource offered by the anastomosing branches on interruption of the principal trunks, and some other physiological facts of great interest, might have been introduced with great propriety; but, neither here, nor in the other parts of the work, can we discern the hand of a master, but only of one who performs well within prescribed limits, but does not venture beyond them.

[To be continued.]

---

*The Rules of the extended Medical Institution for the Benefit of the Sick and Drooping Poor, with an Explanation of its peculiar Design, and various necessary Instructions. By* THOMAS BEDDOES, M. D. pp. 78. Bristol, 1803.

IF this little tract were drawn up by a common hand, and contained only the usual rules and regulations of a charitable institution, we should not have introduced it into our Retrospect; but there is something so truly original in every thing that comes from the pen of Dr. Beddoes as always to fix the attention, at least, of his readers, and not seldom to ensure their warm approbation. It is particularly in large and extended views of the objects of his profession that the author exhibits his talent for deep observation, and nice insight into the characters of different ranks of society;—as a guardian of public health his abilities appear eminently conspicuous.

The purpose of this Institution is singular, it is for the prevention of some of the most widely wasting diseases to which the inhabitants of this country, particularly the hard working, needy, and suffering part of the community are exposed, partly by the pressure of always urgent necessity, partly by prejudice and ignorance in subjects connected with the preservation of health, partly by their own folly, thoughtlessness, and vice, and very largely by the bitter inheritance of the seeds of disease, commonly the only inheritance derived from their progenitors. The purpose of the institution is thus explained by the author.

"Now, then, in what does it consist?—If I am to answer generally, I should say it is—in proposing to check the canker of disease as soon as it fastens on the frame, and to root it out:—the moment any one seems, before his season, inclining towards the grave, to stretch out a helping hand, rear him upright, and set him firmly upon his footing again—Do I speak plainly enough, or shall I add, that it is our purpose to preserve those that suppose themselves only a trifle out of sorts, but are really falling into some deadly disorder,—to fill the feeble with strength to go through the business of their station—to give those, whose weakly childhood marks them out as likely to be cut off in youth, a fair chance for a long and healthy life—in fine, not only to stop short the course of some maladies, but to render the constitution less accessible to them, and to stir up in fathers and mothers an universal spirit of watchfulness over the condition of their tender offspring."

A most

A most forcible appeal is made to the feelings, and, above all, to the common sense, of those to whom this is addressed, by strong portraits of these diseases. We shall present some of these to our readers, not to add to *their* information, but as specimens of this kind of graphical painting.

"Consumption, then, has the harrowing chills and scorching heats of fevers in general. It has the drenching and draining sweats of ague in particular. It has the insupportable languor of various slow disorders. It has the oppression of asthma; indeed, a worse oppression still, since it often grows stronger and stronger, up to actual drowning by inches, or suffocation from matter which there is not strength to bring up. Belonging to the advanced stage of the disease, there is a scene which I have sometimes witnessed, and which I doubt not but others have witnessed also. It is, when the consumptive expire under increasing shortness of breath. They have a sort of agony that will last for hours and even for days with some intermissions. The sufferer gasps; at times he snaps convulsively at the air; and calls, with dismal shrieks, upon the by-standers for Breath! Breath!

"Besides this, there is the violent cough, producing frequent retchings, and even the throwing up of almost every meal for days together. Should the patient at first feel no pains about his chest, he is seldom so lucky as to get into the grave without being visited by them severely,—and by pains elsewhere too. What is most miserable is, that these distresses commonly go on increasing with the inability to bear them. New evils spring up from time to time. Think to what a pitch wretchedness must have risen, when the galled state of the skin renders it almost impossible to find a posture for reposing the weary limbs. After a long siege has been laid to life, attempts are made to seize it by storm. Sudden conflicts arise, and severe conflicts they prove. The first are got over perhaps, though with difficulty. The pitying friends congratulate themselves. But how can one join in their joy, when the struggle is still to be gone over again; and yield at last? I say nothing of the outward change—how the features shrink—the nose sharpens—the eyes grow hollow—the cheek bones project, till we have a living death's head just covered with skin; and one of the most lovely, perhaps, is changed into one of the most pitiable objects the eye of man can behold.

"For it is by no means here as in many other complaints, of which we say—He has youth on his side, and it is odds but he gets over it. In the present case youth is no sort of advantage. Who indeed is ignorant that this dire disorder is most apt to come like a blight over the spring of life? And who can contradict the melancholy observation of the poet?

"Sad Beauty's form foul SCROPHULA surrounds

With bones distorted and putrescent wounds;

And, fell CONSUMPTION, thy unerring dart

Wets its broad wing in Youth's reluctant heart.

"You

"You are, I hope, beginning by this time to conceive what sort of a complaint it is, which I would fain show you in something like its true colours. Very different no doubt it must be from what you had imagined, if you have only passed some poor pale female, basking like a withered lilly in the sun, and have not been used to hold her head as she strains with coughing, or to watch all night by the side of her reeking bed."

Still the reader may not be aware of the precise object of this institution, nor of the method in which the physician is to set about fulfilling its duties. Of this the author gives some farther explanation.

"Upon examining a particular child, it has occurred to me, many scores of times, to ask the parent: Have you any more children? "Yes." I am sure, from what I see about this child, that the rest cannot be healthy. "They none of them complain." Be so good as bring them however such a day. I particularly wish to examine them, and satisfy myself.

"In consequence of this conversation the children are brought. I single out one among the number, and finding various distinct marks of indisposition about him, I enquire: Can you possibly consider this child as in a proper state of health? The parent says again, as he did at first: "I do not hear him complain; and he eats heartily enough; but his meat does not seem to do him any good. He has been getting thinner and weaker these some months." That is speaking to the purpose. Can you suppose a child is as he ought to be, when his food does him no good, and he gets thinner and weaker? I could add several other reasons, if they were such as you could properly understand. But I suppose you will be satisfied if I promise to give him something that shall cause his food to do him good, and make him fuller of flesh and stronger. You must do your part: give him the medicine regularly, and feed him in the best way you can."

Of the fortune or success of such an institution more than doubts will be entertained by many of those who have had the most experience in these subjects; but the efforts of enlightened physicians to improve the health, comforts, and with them the morals of the lower classes of the people survive the fate of individual establishments, and are seldom lost upon that part of society, whose good opinion is the most to be valued and cherished.

